# 

**Published:** 1869-09

**Authors:** 


*Delay of the September No. of the Journal.*—This number has been delayed beyond the usual time of issue in order that we might be able to present our readers with a full and complete account of the proceedings of the first meeting of the "Southern Dental Association." Had we gone to press earlier we should have been compelled to publish a mere outline of the proceedings, such as have appeared in the Sept. Nos. of other Journals. The report we publish is such an interesting one, that we feel satisfied our subscribers will pardon the delay.

